# Lichen Striatus Albus–A Case Report of a Rare Disease Entity

**DOI:** 10.3390/diagnostics15182367

**Published:** 2025-09-17

**Authors:** Beata Zagórska, Jakub Żółkiewicz, Wojciech Biernat, Michał Sobjanek, Martyna Sławińska

**Affiliations:** 1 Department of Dermatology, Venerology and Allergology, Medical University of Gdansk, Mariana Smoluchowskiego 17, 80-214 Gdańsk, Poland; beatazagorska@gumed.edu.pl (B.Z.); zolkiewicz@gumed.edu.pl (J.Ż.); michal.sobjanek@gumed.edu.pl (M.S.); 2 Department of Pathomorphology, Medical University of Gdansk, 80-214 Gdańsk, Poland; wojciech.biernat@gumed.edu.pl

**Keywords:** lichen striatus albus, dermoscopy, histopathology features

## Abstract

We describe the case of an 11-year-old patient who was consulted at the Dermatology Clinic due to a linear hypopigmented skin lesion located on the right arm. The lesion appeared in the first year of life. Based on the clinical presentation, lichen striatus albus (LS) was suspected, which was later confirmed by histopathological examination. LS albus is a rare entity. The condition is usually self-resolving and does not require treatment.

**Figure 1:**
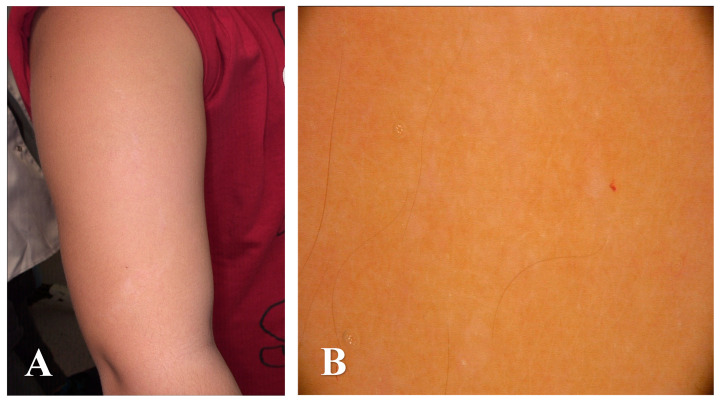
An 11-year-old boy was consulted at the Dermatology Clinic due to a linear hypopigmented skin lesion with the presence of tiny flesh-colored papules distributed along the lines of Blaschko located on the right arm (**A**). The lesion appeared in the first year of life. History of chronic diseases, medications taken, and family history of skin diseases was negative. Dermatoscopy revealed white structureless areas corresponding with an area of linear hypopigmentation and subtle white scale corresponding with the presence of tiny flesh-colored papules distributed along the lines of Blaschko ((**B**), FotoFinder Vexia Medicam 800 HD; 20× magnification; non-polarized light; immersion: ultrasound gel).

**Figure 2:**
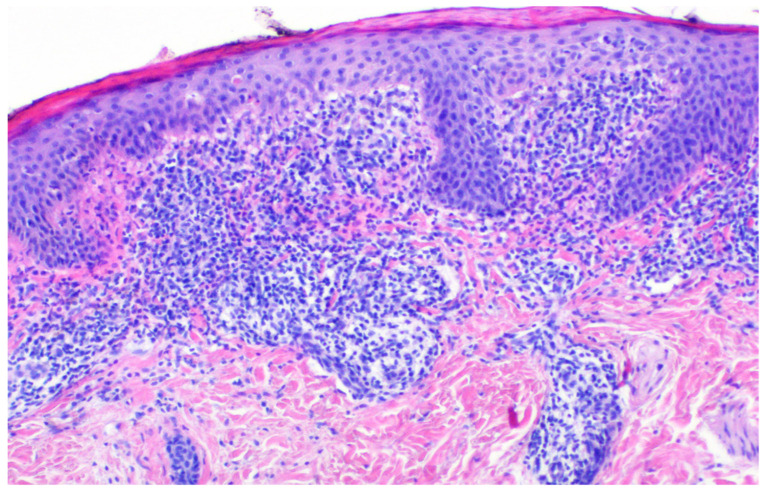
The patient was qualified for a diagnostic biopsy. After obtaining written consent from the patient’s legal guardians, a skin fragment was collected for histopathological examination using a 4 mm punch under sterile conditions. Microscopic results revealed a superficial infiltrate of small lymphocytes focally reaching the epidermis, which shows discrete parakeratosis and single dyskeratotic keratinocytes. Lichen striatus is a rare, benign childhood dermatosis. The condition is more common in girls and is characterized by pink or flesh-colored lichenoid papules that form bands along the lines of Blaschko [1]. Dermoscopically, linear white scales are usually observed, which coincide with the observed clinical features [2]. Hypopigmented macules and/or papules are typical of a rare variant of the disease–lichen striatus albus, which is more often diagnosed in patients with a dark skin phototype. The lesions are most frequently localized to one side of the body, particularly on the extremities [3]. The exact etiology of lichen striatus has not yet been determined [1,2]. Previous studies have suggested a possible link between the disease and infections, trauma, vaccinations, a personal or family history of allergic diseases and treatment with medications like etanercept and adalimumab [4,5]. Differential diagnosis of lichen striatus includes blaschkitis, inflammatory linear verrucous epidermal nevus (ILVEN), linear lichen planus, linear scleroderma, and hypomelanosis of Ito [5,6,7]. Definitive diagnosis is possible based on histopathological examination. The most important histopathological findings of LS and its clinical mimickers are presented in Table 1. The disease usually resolves spontaneously and does not require treatment [1].

**Table 1:** Histopathological features of lichen striatus and its clinical mimickers.

	Lichen Striatus	Blaschkitis	ILVEN	Linear Lichen Planus	Linear Scleroderma	Hypomelanosis of Ito
Epidermis	Mild acanthosis, focal parakeratosis, hyperkeratosis; basal vacuolization and Civatte bodies may be present [3].	Spongiosis (more pronounced compared to LS); mild acanthosis; parakeratosis possible [8,9].	Alternating areas of hypergranulosis with orthokeratosis and agranulosis with parakeratosis (“checkerboard” pattern); psoriasiform hyperplasia [10,11].	Hypergranulosis, wedge-shaped hyperkeratosis, irregular acanthosis, saw-tooth rete ridges; Civatte bodies may be present [12].	Usually unremarkable or mild atrophy [13].	Reduced melanocyte density in the basal epidermis. Decreased number and size of basal melanosomes. Selective reduction in eumelanin. Increased Langerhans cell presence in depigmented areas [14].
Dermis	Dense, band-like lichenoid infiltrate at the dermoepidermal junction (DEJ) (similar to LP) associated with perivascular and periappendagealinflammatory infiltrate–involvement of eccrine glands and ducts distinguishes LS from other lichenoid dermatosies [3].	Less lichenoid pattern; minimal eccrine involvement [8,9].	Mild superficial perivascular infiltrate; less prominent than in LS [10,11].	Dense, band-like lichenoid infiltrate along the DEJ; involvement limited to the upper dermis [12,15].	Thickened, hyalinized collagen bundles; loss of adnexal structures; perivascular lymphocytes [13].	Absence of extracellular melanin (no signs of pigment leakage). No histological signs of inflammatory infiltration [14].

## Data Availability

All relevant data are within the manuscript.
